# Established and emerging roles for ultrasound enhancing agents (contrast echocardiography)

**DOI:** 10.1002/clc.23924

**Published:** 2022-10-02

**Authors:** Bruno Cotter, Ajit Raisinghani, Anthony N. DeMaria

**Affiliations:** ^1^ Division of Cardiology, Department of Medicine, Sulpizio Cardiovascular Center University of California San Diego San Diego California USA

**Keywords:** contrast agents, echocardiography, left ventricular opacification, myocardial perfusion, ultrasound enhancing agents

## Abstract

The ability to opacify the left ventricle and delineate the endocardium after intravenous injection of microbubble ultrasound enhancing agents is of established value to quantify volumes and function in suboptimal unenhanced images, particularly in stress echocardiograms. However, applications other than quantitation of left ventricle structure and function exist for contrast enhanced left ventricular opacification. Contrast agents enable recording of Doppler velocity signals in patients with poor ultrasound transmission, providing estimates of aortic stenosis gradient and pulmonary artery pressures. Contrast echo is of value in detecting apical hypertrophic cardiomyopathy and accompanying apical aneurysms. Most importantly, ultrasound enhancing agents can identify apical and left atrial masses when they cannot be visualized in unenhanced images, and can distinguish thrombi from tumors by visualizing the vascularity inherent in tumors. Contrast agents distinguish trabecular from compacted myocardium in noncompaction syndrome, and hypertrabeculation with other abnormal conditions. A major potential application of ultrasound enhancing agents is myocardial opacification, which can assist in identifying nonviable myocardium. Also, the delayed reappearance of myocardial perfusion after microbubble destruction identifies impaired contrary flow and can diagnose coronary stenosis. Innovative applications of ultrasound contrast agents currently under investigation, include visualizing the vaso vasorum to identify plaques and assess their vulnerability, and theranostic agents to deliver drugs and biologists and to assist in sonothrombolysis. It is anticipated that the role of ultrasound contrast agents will continue to increase in the future.

## 1 INTRODUCTION

1.1

C. Richard (Dick) Conti was an “impact player.” He made an impact on everything that he engaged in, changing it for the better and leaving his imprint. He was a superb clinician, productive clinical investigator, gifted teacher, and effective administrator. His influence upon the University of Florida, the American College of Cardiology, and of course on Clinical Cardiology is easily visible. He succeeded one of us (AND) as President of the American College of Cardiology. We worked closely together, traveled extensively, and shared many experiences, some serious and some humorous. His advice was always sage and of great value. Dick was a giant intellect, an indefatigable worker, and a “tell it like it is” person; it was impossible not to like him. It is truly our honor to have the opportunity to contribute a manuscript to this issue in his memory.

## 2 HISTORY

The first significant clinical description of contrast echocardiography is usually credited to Drs. Gramiak and Shah who in 1969 traced the cloud of echo signals produced by the left atrial injection of indocyanine green as it opacified the left atrium and subsequently the aorta by m‐mode echocardiography.^1^ Subsequently, contrast echocardiography consisted primarily of the injection of microbubbles produced by agitating saline which opacified the right ventricle but not the left since they were removed by the lungs. The recognition that a room air microbubble of the size of a red blood cell would persist intact in circulation for less than a second stimulated the development of microbubbles using heavier molecular weight and less diffusible gases surrounded by a shell comprised largely of albumen lipoproteins. These enhanced microbubbles efficiently persisted in circulation to cross the lungs and opacify the left sided cardiac chambers. They effectively and accurately delineate the endocardial border, an ability of paramount importance in patients with technically suboptimal images (Figure and video 1). There are currently a number of ultrasound enhancing agents approved for this application (Table 1) and the safety of the procedure has been well established. Left ventricular opacification with ultrasonic contrast agents has achieved an established role in clinical echocardiography and is currently applied routinely on a daily basis to define the endocardial border and assess left ventricular size and contraction. However, additional applications of ultrasonic contrast agents have been identified, and several potential applications are currently under development for standard application in clinical cardiology (Table 2).

**Figure 1:**
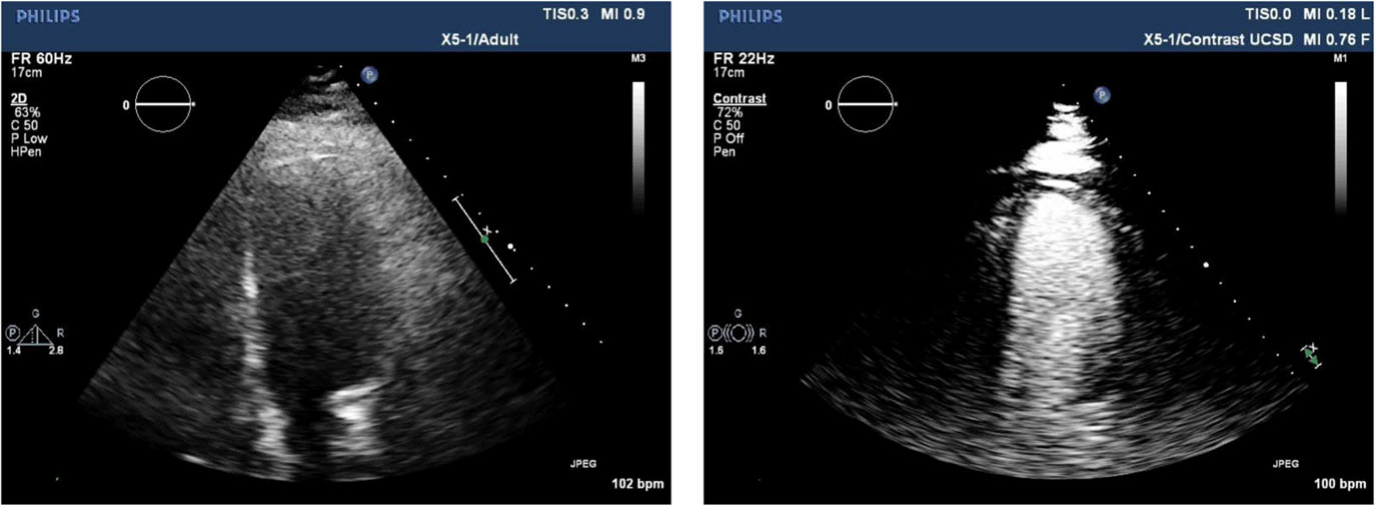
Left Panel: the unenhanced 4‐chamber echo in a patient with a technically suboptimal image. Right Panel: delineation of the endocardial border after left ventricular opacification with contrast.

**Table 1:** Contrast agents

Agent	Mean size (u)	*Gas*	*Shell*
Levovist	2−3	Air	(Galactose)
**Optison**	4.7	Perflouropropane	Albumin
**Definity**	1.5	Perflouropropane	Phospholipid
Imagent	5.0	Perflourohexane‐N	Surfactant
**Lumason (Sonovue)**	2.5	Sulfur hexaflouride	Phospholipid
*Cardiosphere*	4.0	*Nitrogen*	*Polymer*
*Acusphere*	2.0	*Perflourocarbon*	*Polymer*

**Table 2:** Contrast echo other than border definition

Doppler enhancement Apical hypertrophic cardiomyopathy Cardiac masses Tumor versus clot 3D enhancement Noncompaction Myocardial perfusion Vascular enhancement

## 3 LEFT VENTRICULAR BORDER DEFINITION

As previously stated, ultrasound enhancing agents, referred to in the vernacular as contrast agents, have achieved an established role for left ventricular opacification and identification of the endocardial border. A number of studies have demonstrated the ability of contrast enhanced echocardiograms to yield measures of left ventricular volume and ejection fraction that correlate well with other techniques such as magnetic resonance imaging in patients in whom the unenhanced echocardiogram is technically inadequate.^2^, ^3^ Thus, administration of contrast agents can successfully identify the endocardial border in patients in whom endocardial targets cannot be imaged with unenhanced images. Of perhaps greatest importance, left ventricular opacification with ultrasound enhancing agents has been documented to be capable of impacting the management of patients with known or suspected cardiovascular disease. In a landmark study, Kurt and associates initially informed referring physicians of the results of echocardiograms in patients with technically inadequate studies, and then subsequently informed them of the results after contrast enhancement. They then compared the ultimate management of those patients to that which was initially planned based upon the inadequate recording. They found that the referring physicians changed management by altering or eliminating additional procedures or changing medications or both in one‐third of the patients included. These data established the fact that contrast echo not only resulted in superior images, but that those images had information content sufficient to alter medical management.^4^

Ultrasound enhancing agents may also be of value in defining endocardial landmarks on three‐dimensional echocardiography which is less sensitive in imaging low intensity targets than conventional two‐dimensional imaging.

Despite documentation that ventricular opacification by contrast echo could accurately identify left ventricle volumes and ejection fraction and thereby influence patient management, implementation of these recordings in echo laboratories has been slow and variable. Evidence exists to indicate that, although 15%−30% of echocardiographic studies are marginal or inadequate, especially stress echo and those performed in the intensive care unit, only approximately 5% of echo studies employ ultrasound enhancing agents.^5^ A number of factors appear to contribute to this underutilization, such as the need to do an intravenous injection and the legacy mindset of interpreting echocardiograms for years without the benefit of contrast visualization. This has led to an attempt to define those patients in whom ultrasound enhancing agents should be applied in their echocardiogram Obvious candidates include patients with obesity, chronic obstructive pulmonary disease, chest deformities, and any patient in whom acoustic windows are limited to yield adequate images. An important application of left ventricular opacification has been to increase the reproducibility of echo quantification of left ventricular size and contraction. It has been demonstrated that the use of ultrasound enhancing agents and markedly reduced variability in echo quantification and yield measurements of echocardiographic size and function to reproducibility is comparable to that of other techniques such as magnetic resonance imaging.^6^, ^7^ Based upon these data, the guidelines for contrast utilization of the American Society of Echocardiography recommend the use of ultrasound enhancing agents in all patients undergoing rest echocardiograms for the indication of evaluation of left ventricular systolic function, not just those in whom images are technically inadequate.^2^ The criteria for determining that studies are technically inadequate include the inability to visualize the endocardial border in two or more contiguous segments of the left ventricle in unenhanced images. Obviously, since abnormal contraction of even one left ventricular segment on a stress echocardiogram establishes an abnormal study, the indication for contrast enhancement in this setting is greater.

While the ability to enhance endocardial border definition by contrast echo is well established and integrated into practice, a number of other applications other than border definitions are often encountered. The injection of microbubbles produced by agitated saline to identify right to left cardiac or intrapulmonary shunts is well established and will not be further discussed.

## 4 ENHANCEMENT OF DOPPLER RECORDINGS

**Figure 2:**
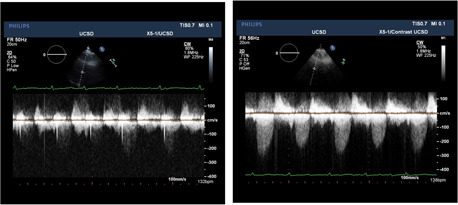
Left Panel: the aortic stenosis jet recorded from a patient with poor ultrasound transmission. Right Panel: the aortic stenosis jet after contrast injection; a jet velocity of approximately 250 cm/s is seen.

**Figure 3:**
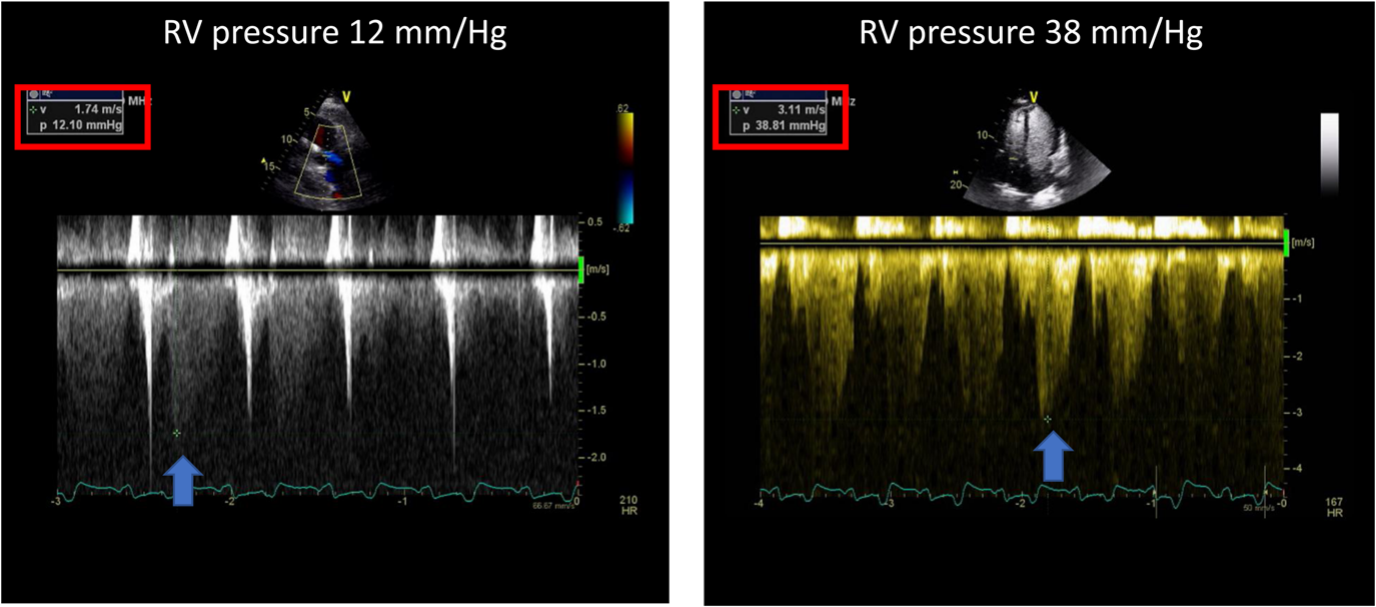
Left Panel: tricuspid regurgitant jet velocity with a suboptimal resting echo yielding an estimated right ventricular pressure of 12 mm/Hg. Right Panel: after contrast enhancement a tricuspid regurgitant jet in excess of 3 m/s is recorded yielding a right ventricular pressure of 38 mm of mercury.

The injection of contrast agents can be utilized to enhance Doppler recordings of flow in the central and peripheral circulation in patients with suboptimal studies. Accurate measurement of transvalve velocities from Doppler velocity recordings has played a major role in the ability of echocardiography to eliminate the need for cardiac catheterization. The initial application of this technique was in the setting of aortic stenosis (Figure 2), where it was demonstrated that transaortic valve pressures derived from contrast enhanced Doppler velocity measurements in technically inadequate unenhanced examinations correlated very well with those obtained by direct intracardiac pressure measurement.^8^ The ability of echocardiography to provide estimates of pulmonary artery pressure is also well ensconced in clinical cardiology, and identification of the envelope of the tricuspid regurgitant jet by contrast enhancement has been shown to yield accurate estimates of pulmonary artery pressure in patients in whom standard recordings are technically limited (Figure 3).^9^ Spectral Doppler recordings of pulmonary vein flow may be of significant value in patients undergoing cardiac ultrasound, and are often technically difficult to visualize. Contrast echo can yield well defined pulmonary venous Doppler tracings even in difficult to examine patients.

## 5 APICAL HYPERTROPHIC CARDIOMYOPATHY

An important use of left ventricular opacification has been the diagnosis of apical hypertrophic cardiomyopathy. It is often impossible to delineate the endocardial border of the apex in patients in whom examinations are technically difficult. The administration of contrast can provide such identification and thereby diagnose apical hypertrophic cardiomyopathy if present (Figure and video 4). In fact, many cases of hypertrophic cardiomyopathy are identified serendipitously when injecting ultrasound enhancing agents to assess wall motion abnormalities in patients with chest pain or abnormal electrocardiograms. The injection of contrast may be of value even in patients in whom apical hypertrophy is visible (Figure 5) to detect the presence of an apical aneurysm which often occurs in this setting and has adverse prognostic significance. Finally, regarding hypertrophic cardiomyopathy, the intracoronary injection of contrast agents is an integral part of performing the alcohol‐septal, ablation procedure to treat this condition. The opacification that occurs following injection into a septal perforator artery outlines the area of muscle that will be destroyed with the injection of alcohol into that branch. Obviously, it is important that the septal artery to receive alcohol injection supplies the area of septal hypertrophy which is the target for ablation.

**Figure 4:**
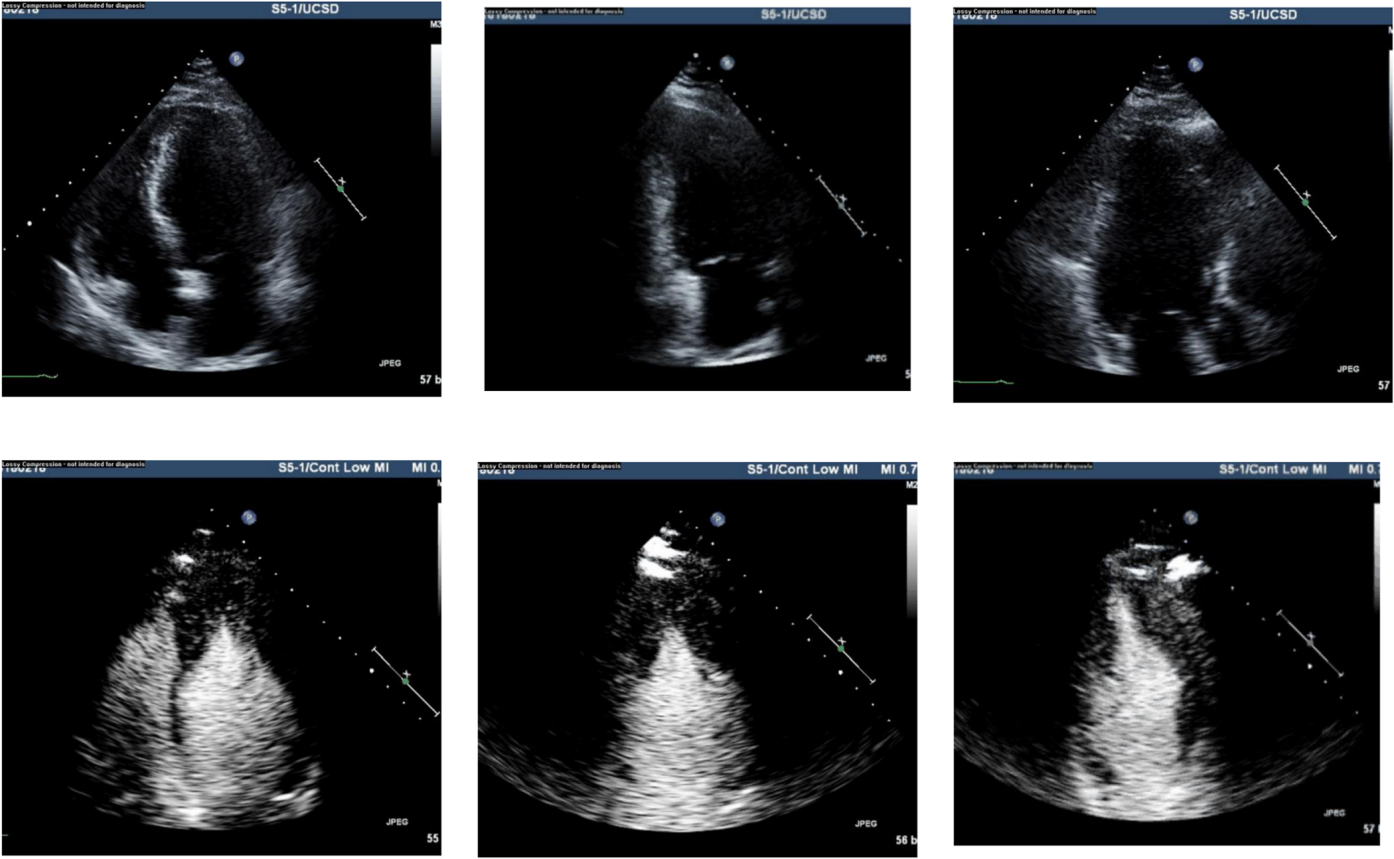
The upper panels show the 4‐chamber (left) 2‐chambers (center) and 3‐chamber (right) unenhanced echo images in a patient with hypertrophic cardiomyopathy. Bottom panels demonstrate a clear delineation of the apical hypertrophy in all three individual views after contrast injection.

**Figure 5:**
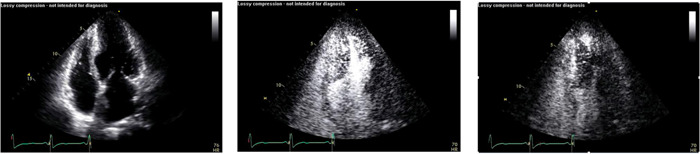
A patient with apical hypertrophic cardiomyopathy which is not well delineated in the unenhanced apical 4‐chamber view (left), well delineated following contrast (center), and demonstrates an apical aneurysm in mid systole (right)

## 6 CARDIAC MASSES

**Figure 6:**
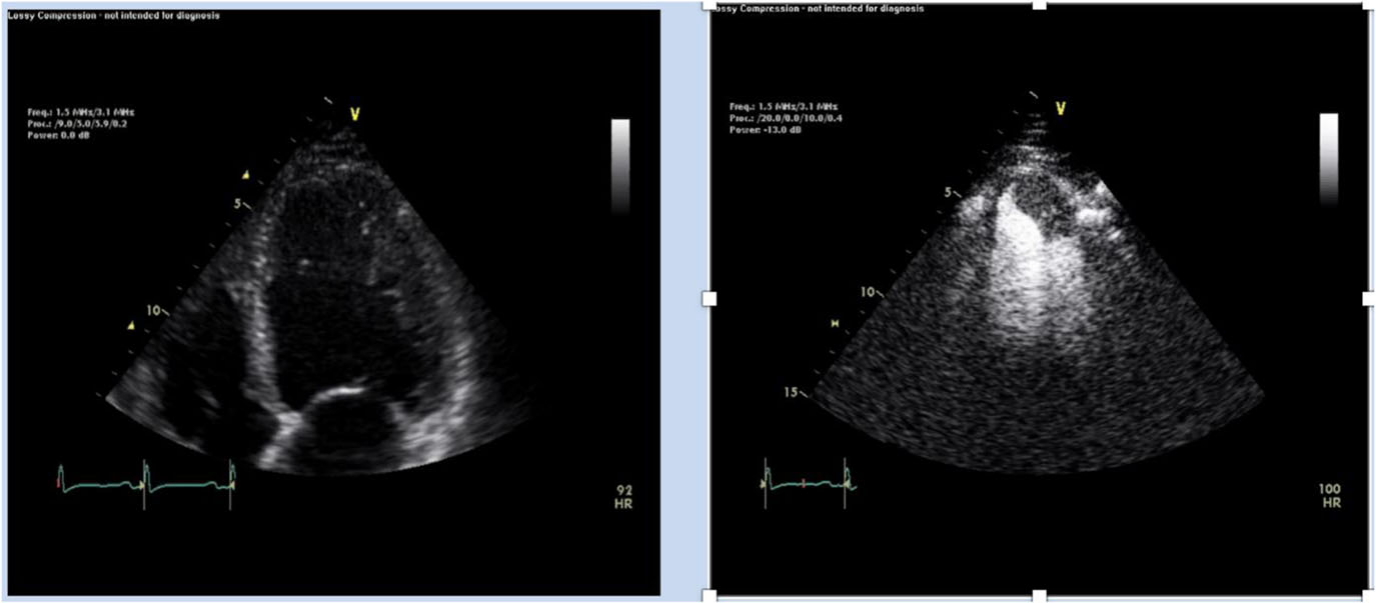
Left Panel: the apical 4 chamber recording before contrast injection while the right panel clearly demonstrates an apical thrombus following administration of an ultrasound enhancing agent

Probably the major application of ultrasound enhancing agents derived from left ventricular opacification other than border delineation is the identification of cardiac masses, particularly left ventricular thrombi. Numerous examples exist of patients with sizable and protruding left ventricular thrombi that are not adequately visualized by unenhanced images that are well delineated after the administration of contrast (Figures 6).^10^ The size and mobility of a thrombus has been associated with the propensity for embolization, and contrast enhancement can assist in determining these characteristics. Anticoagulation with vitamin K antagonists has been the traditional treatment, although recent studies that direct activing oral anticoagulants may be equally effective. Although routine screening with ultrasound enhancing agents of patients with severe left ventricular enlargement and dysfunction has been found to yield thrombi that went otherwise undetected, this strategy has not yet proven to be cost effective. Contrast agents may also be of value in the detection of thrombi in the left atrial appendage. While clots are generally well visualized by transesophageal echocardiography, ultrasound enhancing agents may be of great value in distinguishing artifacts and pectinate muscles from true thrombi.^11^

**Figure 7:**
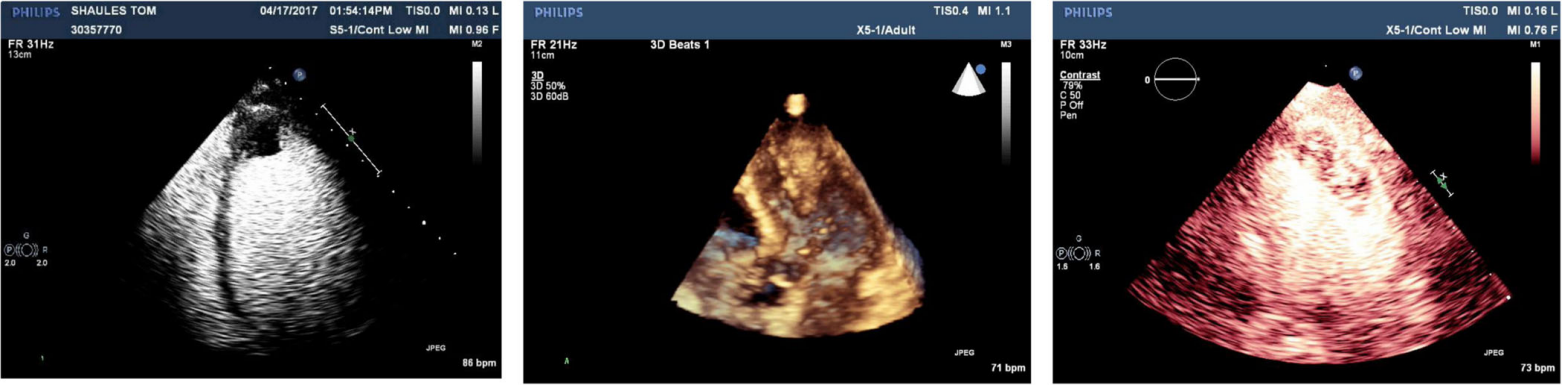
Left panel delineates an apical thrombus that is free of any ultrasound reflectances. Middle panel show the echo of a patient with a large apical tumor that contains reflectances generated by tissue in homogeneity. Right panel demonstrates the same tumor after contrast agent administration which clearly delineates the persistent vasculature.

Cardiac tumors are less frequently encountered on echocardiogram than thrombi and are usually well detected even without the administration of contrast agents. However, contrast agents can be of value in distinguishing tumors from thrombi. Tumors are usually well vascularized while thrombi are not, typically yielding sonolucent thrombus images versus tumors with scattered reflectances (Figure 7). However, the use of contrast agents provides the optimal distinction of tumors from thrombi. Specifically, administration of an ultrasound contrast agent will enhance the vascular bed of a tumor but not a thrombus. It has been demonstrated that tumors will manifest an ultrasound intensity comparable to myocardium after the injection of contrast, whereas thrombi will not.^12^ Thus, ultrasound enhancing agents often yield additional information even when a mass is clearly visualized in the left ventricle.

## 7 HYPERTRABECULATION AND NONCOMPACTION CARDIOMYOPATHY

**Figure 8:**
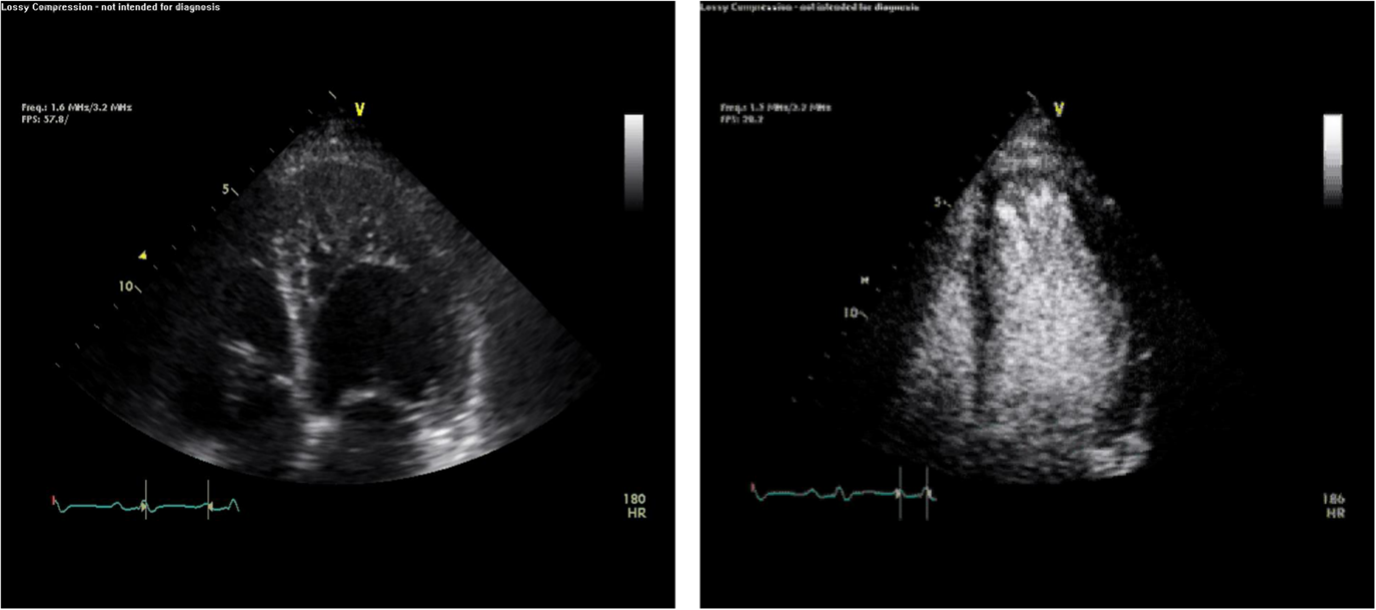
Left Panel demonstrates a marked hypertrabecular pattern in a patient with noncompaction while the right panel illustrates that contrast fills the intrabecular clefts delineating the compacted myocardium

**Figure 9:**
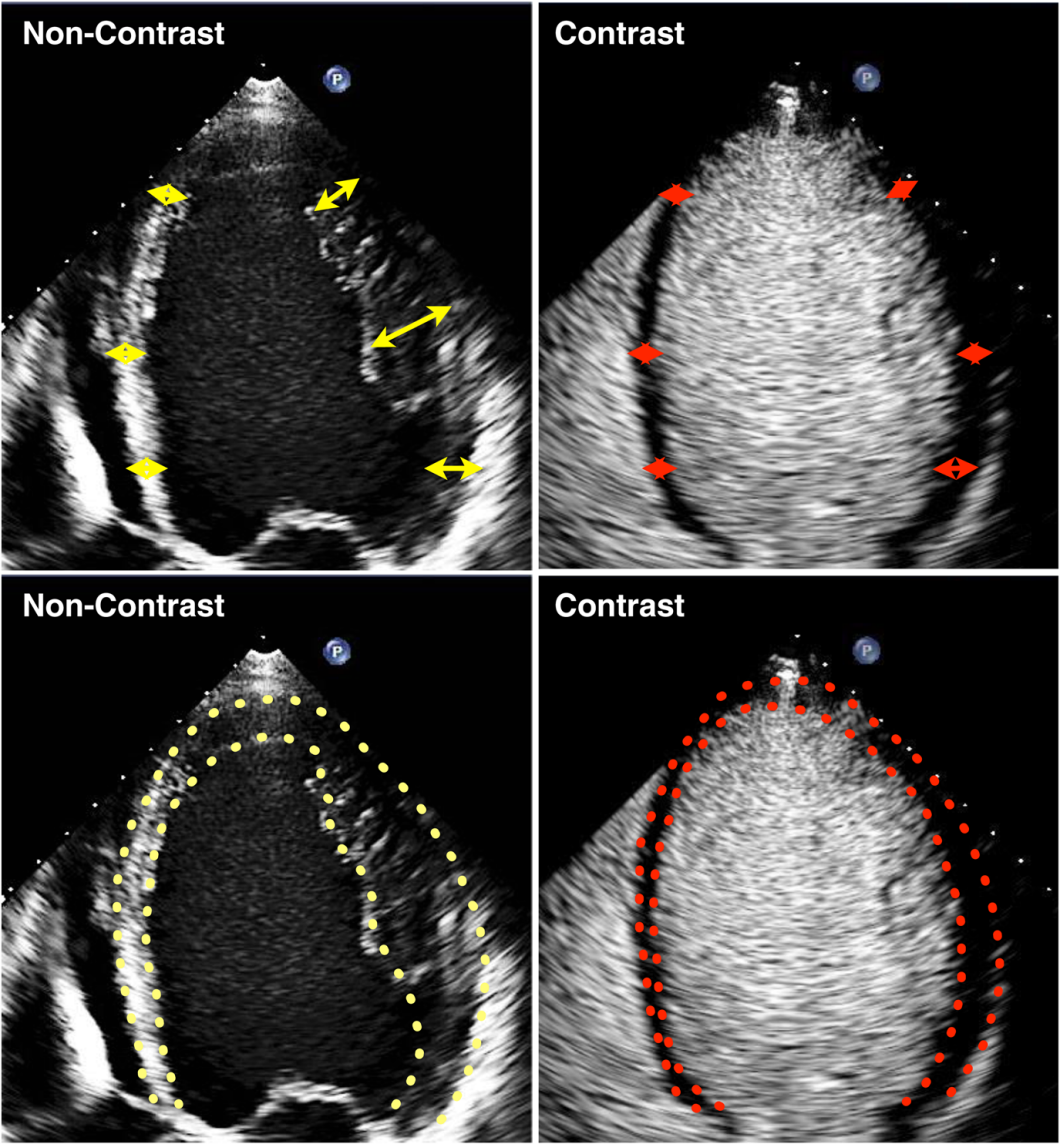
Left panels show the unenhanced four chamber view in a patient with a dilated cardiomyopathy before contrast enhancement while the right panel administration of enhancing agents shows the thin compacted myocardium and that the apparent endocardial border of the ventricle consists of hypertrabeculation

Noncompaction cardiomyopathy is an uncommon congenital abnormality.^13^ Embryonic trabeculae are typically remodeled during gestation as left ventricular volume compresses intrabecular clefts to yield a compacted myocardium. Noncompaction cardiomyopathy occurs when this process does not occur. Contrast echocardiography can confirm the diagnosis by identifying the compacted myocardium and demonstrating that its thickness is approximately half of that of the distance of the epicardial signals to the tips of trabeculated myocardium (Figure 8). Other conditions such as ischemic and nonischemic dilated cardiomyopathy can also result in expansion of intertrabecular clefts and reduction of compacted myocardium, and these can also be easily confirmed by contrast echo. This has led to the concept of hypertrabeculation as a pathomorphology for which noncompaction syndrome is only one etiology (Figure 9).^14^, ^15^ Studies have demonstrated that contrast identification of hypertrabeculation and decreased compacted myocardial thickness has adverse prognostic significance in patients with left ventricular dilation and advanced dysfunction.^14^

## 8 MYOCARDIAL CONTRAST ECHOCARDIOGRAPHY

**Figure 10:**
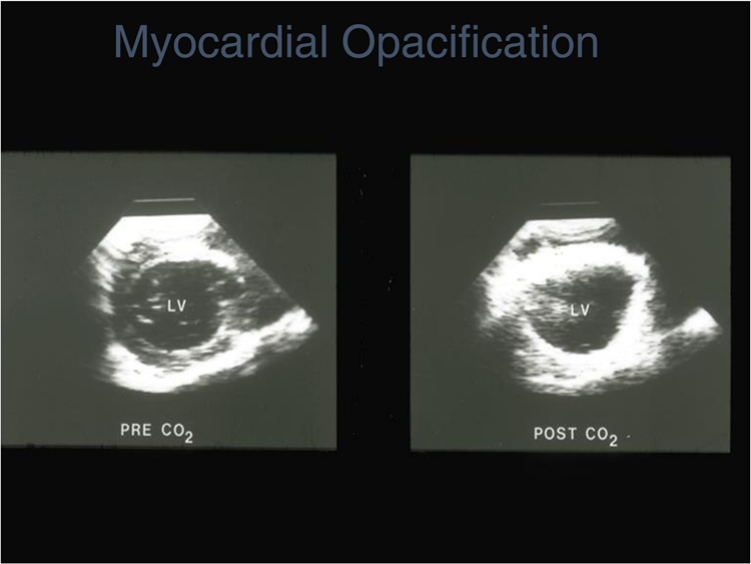
Images from a preclinical study in the parasternal short axis view before (left) and following (right) the intracoronary injection CO_2_

**Figure 11:**
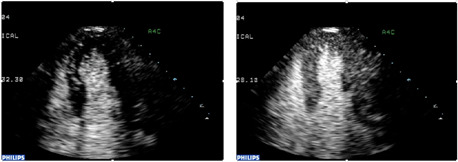
The myocardial contrast echocardiogram obtained in a normal subject. Left panel is taken at the arrival of left ventricular contrast while the right panel shows myocardial opacification delineating myocardial perfusion.

**Figure 12:**
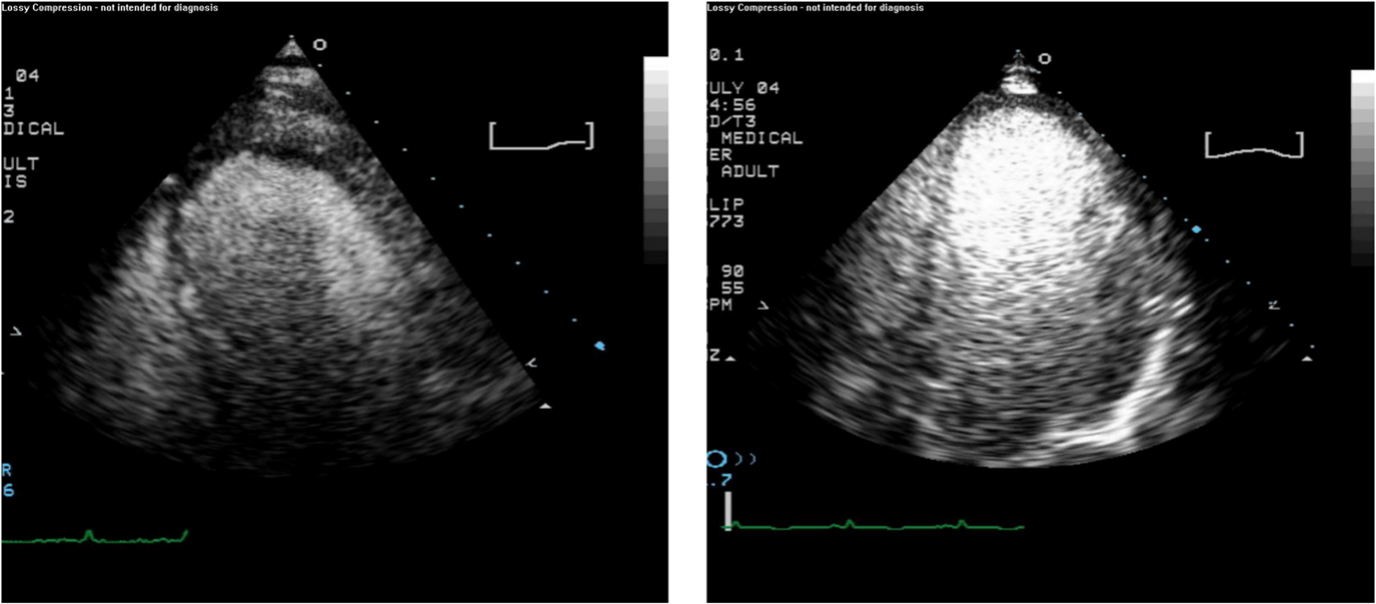
Left panel demonstrates myocardial contrast echocardiogram in a patient with an anterior myocardial infarction in whom virtually no apical opacification is seen indicative of nonviable myocardium. The panel on the right shows another patient with an anterior apical myocardial infarction in whom at least partial apical opacification exists suggesting myocardial viability.

Preclinical studies initially demonstrated that the direct intracoronary injection of CO_2_ was capable of intensely opacifying the myocardium, thereby providing the potential to assess myocardial perfusion and coronary blood flow^16^ (Figure 10). In fact, intracoronary contrast injection of microbubbles from agitated radiographic contrast agents were of great importance to defining the no‐flow phenomenon following coronary recanalization.^17^ However, significant technical advances were required to achieve opacification of the myocardium following intravenous injection. It was recognized that the transmission of a low energy ultrasound beam could resonate contrast bubbles to enable visualization, and that progressively higher energy pulses could result in second harmonic signals and then ultimately destroy the contrast agent. Myocardial opacification by intravenous contrast agent injection was ultimately achieved using relatively low energy ultrasound transmission and imaging schemes to distinguish the agent from myocardium (Figure 11). Even with these advances the yield of high‐quality contrast myocardial perfusion images has continued to be lower than those of left ventricular opacification. Importantly, it was observed that following the destruction of contrast microbubbles by a high energy ultrasonic pulse, the time course of reappearance of myocardial opacification served as the basis for estimating coronary blood flow and volume.^18^ Moreover, a decrease in the refilling time of a myocardial segment after contrast destruction in destroy/refill sequences was found to be a marker of coronary artery disease. In this regard, contrast echocardiography provided analogous information regarding myocardial perfusion to radionuclide and magnetic resonance techniques. Contrast myocardial perfusion studies have been demonstrated to be accurate and applied frequently to identify viable myocardium by virtue of opacification produced by persistent microcirculation in a manner similar to radionuclide and magnetic resonance techniques (Figure 12).^19^

Demonstration of myocardial opacification following intravenous injection of ultrasound enhancing agents led to an immediate enthusiasm and anticipation that this methodology could be applied clinically. In fact, several multicenter trials have demonstrated that myocardial contrast echo yields similar accuracy in identifying coronary atherosclerotic stenosis as does radionuclide methods.^20^, ^21^ Nevertheless, these agents have not yet achieved widespread application in clinical cardiology for multiple reasons. Myocardial perfusion images with contrast echo are still often inadequate in difficult to study patients. The pulsing sequences utilized are more complex than routine echo studies, require expertize, and there is no universally agreed upon protocol. Quantitation of myocardial perfusion by contrast echo has limited reproducibility thus far, and only a limited number of multicenter studies have been published. In addition, reimbursement for this study has been slow to come. However, a current procedural terminology (CPT) code under Category 3 has been established for the procedure. Although there is no reimbursement at present for this CPT code, it is the first step in obtaining reimbursement if robust application of the technology and use of the code is documented. Nevertheless, research thus far has clearly demonstrated that myocardial contrast echo studies have the information content necessary to identify abnormal perfusion and coronary stenosis, and the anticipation is that the technique will be refined and utilization will increase as time goes by.

## 9 FUTURE APPLICATIONS

Several innovative applications of ultrasound enhancing agents are currently being investigated. It is well documented that atherosclerotic plaques are often accompanied by proliferation of vasa vasorum on the epithelial surface of the vessel that provide nutrient blood flow to the plaque. It has now been demonstrated that ultrasound enhancing microbubbles can be detected in plaques as evidence of proliferation of the vasa vasorum.^22^ This contrast demonstration of the vasa vasorum is of value not only in the identification of plaques but also as a marker of the risk of the plaque leading to an acute coronary syndrome. Thus far these studies have been directed toward visualizing plaques in the carotid arteries, which are more accessible to imaging than the coronaries. Novel studies are also being directed toward the study of intracavitary blood flow patterns. In particular, the vorticity, energy dissipation, and resonance time of left ventricular diastolic filling have been studied in normal and disease states. Use of ultrasound agents to enhance the recordings of intracardiac flow patterns convey certain advantages and is currently being evaluated for its information content. Contrast agents are also being evaluated for a role as “theragnostic” agents. Specifically, they are being studies as vehicles to facilitate the delivery of drugs and biologics, such as stem cells, and as a modality to assist in the ablation of clots and other tissues.^23^

The use of ultrasound enhancing agents, or contrast echocardiography, has now been utilized in clinical cardiology for 50 years. The primary use of contrast echo to opacify the left ventricle and delineate the endocardial border is well established to be of value and is commonly applied in clinical laboratories. Left ventricular opacification is of proven value in assessing volumes, ejection fraction, and wall motion, and in achieving reproducibility of findings. It has been demonstrated to be capable of altering patient management in those with technically suboptimal recordings. Nevertheless, available data indicate that there is generally an underutilization of ultrasound enhancing agents, even for this well‐established application. There are a number of important applications of left ventricular opacification other than for delineation of the endocardial border. Among them, amplification of Doppler echo and color flow signals, detection of hypertrophic cardiomyopathy, particularly when apical in location, detection of cardiac masses and distinguishing tumors from blood clots, establishing the presence of hypertrabeculation and assisting in the diagnosis of the noncompaction syndrome are critical to the optimal performance of echocardiography. Opacification of the myocardium by intravenous injection of ultrasound enhancing agents is feasible and has the potential to become a first line modality to assess myocardial perfusion. Technical advances will be important to achieving widespread application for this indication. The potential applications of ultrasound contrast agents continue to grow and be explored both for diagnosis and therapy. It is anticipated that the use of contrast agents will continue to play an increasingly important role in the echocardiography laboratory of the future.

## Data Availability

Data openly available in a public repository that issues datasets with DOIs Is for Special Issue: C. Richard Conti Comments to Payment Admin: APC fees are waived for all special issue submissions. C Richard Conti special issue of CLC.
